# Actin stress fibre subtypes in mesenchymal-migrating cells

**DOI:** 10.1098/rsob.130001

**Published:** 2013-06

**Authors:** Tea Vallenius

**Affiliations:** Institute of Biotechnology, University of Helsinki, PO Box 56, Helsinki 00014, Finland

**Keywords:** actin, mesenchymal, migration, dorsal stress fibres, transverse arcs, ventral stress fibres

## Abstract

Mesenchymal cell migration is important for embryogenesis and tissue regeneration. In addition, it has been implicated in pathological conditions such as the dissemination of cancer cells. A characteristic of mesenchymal-migrating cells is the presence of actin stress fibres, which are thought to mediate myosin II-based contractility in close cooperation with associated focal adhesions. Myosin II-based contractility regulates various cellular activities, which occur in a spatial and temporal manner to achieve directional cell migration. These myosin II-based activities involve the maturation of integrin-based adhesions, generation of traction forces, establishment of the front-to-back polarity axis, retraction of the trailing edge, extracellular matrix remodelling and mechanotransduction. Growing evidence suggests that actin stress fibre subtypes, namely dorsal stress fibres, transverse arcs and ventral stress fibres, could provide this spatial and temporal myosin II-based activity. Consistent with their functional differences, recent studies have demonstrated that the molecular composition of actin stress fibre subtypes differ significantly. This present review focuses on the current view of the molecular composition of actin stress fibre subtypes and how these fibre subtypes regulate mesenchymal cell migration.

## 2. A historical glance at actin stress fibres

**Figure 1.**
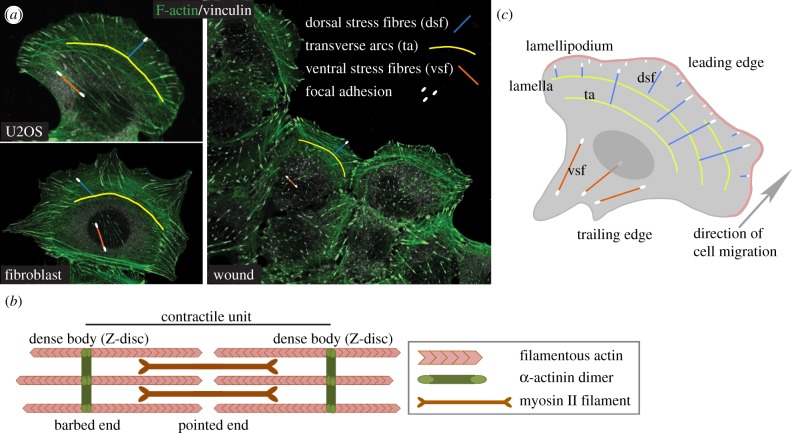
(*a*) F-actin and vinculin co-stained immunofluorescence images demonstrate actin stress fibre subtypes in a migrating human osteosarcoma cell (U2OS), in a fibroblast that recently spread on fibronectin (fibroblast) and on a wound scratch in U2OS cells (wound). Examples of actin stress fibre subtypes and attached adhesions are colour coded as indicated. (*b*) An illustration of the sarcomeric-like stress fibre structure, which exhibits the opposite polarity, i.e. barbed ends of actin filaments are anchored to the lateral ends of each contractile unit (dense body, in muscle cells referred to as Z-disc). Actin filaments that are organized in parallel are cross-linked by α-actinin (green). During contraction myosin II motors (dark red) move towards the barbed ends. (*c*) A schematic of the actin stress fibre subtypes in a mesenchymal-migrating cell. Colour codes and abbreviations are the same as in (*a*). A red curved line at the leading edge represents the branched network of actin filaments at the lamellipodium. Behind the lamellipodium is the contractile lamella.

The present review discusses the role of actin stress fibre subtypes, namely dorsal stress fibres, transverse arcs and ventral stress fibres, in regulating mesenchymal-migrating cells (figure 1). To begin, I will briefly highlight some key historical findings of actin stress fibres to provide a broader perspective on the recent progress in the understanding of the molecular identity and functional significance of stress fibre subtypes.

The historical origins of the term ‘stress fibres’ can be traced back to a study describing cytoplasmic fibres in non-muscle cells cultured on glass substrates in the middle of the 1920s [1]. The authors of this study reasoned that these fibres formed due to the physical stress generated in the cytoplasm as the cells spread out or migrated over glass substrates [1,2]. Currently, this observation is still valid, and in parallel with biochemical signals, physical cues such as substrate rigidity, topography or fluid flow are well established regulators of stress fibre assembly and disassembly [3–5]. Several studies have provided a breakthrough in understanding the function of stress fibres [6–9], for example reporting that these structures actually contained actin in filamentous form. This was first demonstrated using a proteolytic fragment of myosin (S1/heavy meromyosin) that had been previously shown to interact with filamentous actin in muscle cells [7]. Importantly, this study together with subsequent actin immunostaining [10] also provided direct microscopic evidence that actin is a component of non-muscle cells and not just a muscle protein. This evidence motivated a large number of other immunofluorescence-based studies revealing that in addition to actin, actin stress fibres and muscle myofibrils shared several key molecules, such as myosin [11,12], tropomyosin [13] and α-actinin [14]. Over time, it was found that the majority of these shared molecules are actually encoded by a set of muscle- and non-muscle-specific genes that share high sequence identity [15–17]. In parallel with the identification of actin stress fibre components, independent studies indicated that actin stress fibres are contractile [18–22]. These studies involved, for example, experiments in which cell contraction was detected upon the exposure of permeabilized cells to Mg^2+^-ATP [21], the serum stimulation of starved fibroblasts resulted in the shortening of actin stress fibres [22] or a deformable silicone rubber wrinkled while in contact with contractile non-muscle cells [20].

In addition to their molecular composition and contractility, the resemblance between myofibrils and actin stress fibres was noted in terms of actin filament orientation. Earlier muscle studies had demonstrated that single actin filaments exhibited an innate polarity, in which the filament contained a barbed (plus) and a pointed (minus) end. Furthermore, single contractile units (sarcomeres) along the myofibrils contained bundled actin filaments with opposite polarities, indicating that the barbed ends were tethered to the lateral ends of each sarcomere (Z-disc), and the shortening of the unit occurred when the myosin motors moved towards the barbed ends [23] (figure 1*b*). In the late 1970s, several studies emphasized that actin stress fibres also exhibited a sarcomere-like organization of actin filaments [21,22,24,25]. However, a notable dissimilarity was the lack of regular periodicity, which was typical in myofibrils. In addition, some studies reported the existence of a mixed polarity or uniform polarity of actin filament arrays in non-muscle cells [26,27]. Currently, the polarity of the actin stress fibres is still of great interest because it defines the contractile properties of these fibres [28]. In particular, current progress in understanding the role of actin stress fibre subtypes in regulating cell migration has shifted towards an increasing interest in polarity, which is only partially characterized. Taken together, earlier studies on actin stress fibre morphology, molecular composition, contractile function and filament orientation have established the view that these fibres resemble sarcomeric structures in muscle cells. Despite the parallels with muscle sarcomeres, growing evidence suggests that actin stress fibres consist of subtypes, which differ significantly in terms of their molecular composition, contractility and function in migrating cells [28–33].

## 3. Actin stress fibres in migrating cells

In higher eukaryotes, cells can migrate using different migration modes, which are often classified on the basis of the morphology of their migration patterns, cytoskeletal organization and cell–matrix interactions [34–37]. The broad classification of the migration modes involves either single (amoeboid or mesenchymal) or collective (a group of cells) cell migration [34,35]. Examples of cells using single amoeboid or mesenchymal modes of migration include migrating leucocytes and fibroblasts, respectively. Typically, leucocytes have been described as rounded, fast migrating cells (10 µm min^–1^), which loosely attach to the surrounding extracellular matrix (ECM) and squeeze through the ECM gaps [34–36]. In contrast to leucocytes, mesenchymal elongated or fan-shaped fibroblasts migrate slowly (0.1–1 µm min^–1^); they are engaged with the ECM and are capable of remodelling and degrading the matrix while migrating [34,35]. The observed dissimilarities in amoeboid and mesenchymal-migrating cells are largely due to differences in their actin-based structures and adhesion sites. In mesenchymal-migrating cells, widely documented actin-based structures are the branched actin network at the lamellipodium and actin stress fibres (figure 1*c*). Since the 1970s, a substantial number of studies have characterized the model of lamellipodium-based cell movement in fibroblasts [38–42]. This model involves active actin polymerization in the vicinity of the leading plasma membrane (lamellipodium), which results in pushing forces that are required to displace the cell forward (formation of a protrusion). Actin stress fibres can be detected both at the leading lamella and at the trailing edge of mesenchymal-migrating cells, and these fibres are not evident in amoeboid cells [35]. It is noteworthy that leucocytes do not assemble actin stress fibres even when cultured on a stiff matrix, which is a well-characterized physical cue inducing actin stress fibre formation in fibroblasts and epithelial cells [5]. Although it is widely accepted that actin stress fibres promote cell migration, this view has not been always self-evident. At the end of the 1970s and in the beginning of the 1980s, the following issue puzzled researchers: actin stress fibres were observed in migrating cells [22], but they were also increased in non-migrating cells, such as fibroblasts, cultured on either plastic or glass for several days [43]. The abundant actin stress fibres in non-migrating cells together with studies reporting that some cells (such as leucocytes) migrate even faster in the absence of actin stress fibres led some researchers to conclude that stress fibres inhibited cell migration [43]. Subsequently, this conclusion was dispelled, at least partly due to a more accurate understanding of the different migration modes and actin stress fibre differences in migrating and stationary cells. Furthermore, it is important to emphasize that in addition to being stationary or migratory, cells can convert from one migration mode to another. For example, disseminating cancer cells have an incredible capability to change their migration modes and even their cell identity while migrating from a primary tumor to secondary sites (metastasis). Regarding mesenchymal migration, cancer dissemination is a valid example, because a critical event in metastasis often involves the transition between an epithelial cancer cell to a more mesenchymal cell type (epithelial-to-mesenchymal transition), which is thought to provide advantageous properties for cell migration [44,45]. The current view of actin stress fibres in mesenchymal-migrating cancer cells or in normal cells in organ development and tissue regeneration is mainly based on studies performed using two-dimensional cell culture conditions. These studies have provided valuable information on the role of actin-based structures in regulating cell migration. In the remainder of this review, I will focus on the recent advances in our understanding of the significance of actin stress fibre subtypes in regulating mesenchymal cell migration.

## 4. Actin stress fibre subtypes in mesenchymal cell migration

In the late 1990s, actin stress fibres in spreading fibroblasts were subclassified as dorsal stress fibres, transverse arcs and ventral stress fibres on the basis of their different subcellular localization and termination sites [46] (figure 1*a,b*). After some low profile years, a study by Hotulainen & Lappalainen [30] reinvigorated studies in stress fibre subtypes by identifying two distinct actin assembly mechanisms using mesenchymal-migrating cells as a model system. Importantly, the authors used simple markers to distinguish between different fibre subtypes, such as F-actin (phalloidin) and focal adhesion molecules (vinculin). Consistent with the initial classification performed in fibroblasts [46], Hotulainen & Lappalainen [30] found that the leading edge of human osteosarcoma (U2OS) cells contained dorsal stress fibres and transverse arcs, which orientated perpendicularly to each other. In addition, using live cell imaging and photobleaching, this study demonstrated that dorsal stress fibres elongated primarily from leading edge focal adhesions. Presumably, this assembly mechanism resulted in actin stress fibres, which exhibited a uniform polarity, that is, with their constituent filament barbed ends pointing towards the leading edge [28]. This observation is important because uniform polarity markedly differs from the opposite polarity model of sarcomeres. Thus, the uniform polarity of dorsal stress fibres strongly suggests non-contractile properties in dorsal stress fibres. An additional characteristic of dorsal stress fibres is that these fibres are attached to focal adhesions only at the leading edge, whereas the other end often interacts with transverse arcs. This arrangement forms a link between the transverse arc and maturing focal adhesion [30,31,47] (figure 1*c*).

Unlike the straight dorsal stress fibres, curve-shaped transverse arcs do not directly interact with focal adhesions [30,31,46,48]. Data obtained from time-lapse images demonstrated that transverse arcs assemble via end-to-end annealing of short actin bundles generated at the leading lamellipodium [30]. These contractile sarcomere-like fibres, which orientate in parallel with the leading edge, constantly move towards the nucleus and disassemble prior to reaching it. A recent study by Aratyn-Schaus *et al*. [49] focusing on the formation of lamellar actin networks in U2OS cells suggested an alternative model for the formation of lamellar actin stress fibres, which presumably represented the assembly of transverse arcs. In this model, at low forces, the lamellar region of U2OS cells contained a contractile lamellar actin network in the absence of evident actin stress fibres. Upon an increase in force (removal of blebbistatin), myosin II remodels actin into thin bundles. This action promotes the accumulation of α-actinin into these actin–myosin bundles, resulting in the assembly of actin stress fibres. Noted differences between these two models potentially arise from the experimental design [30,49]. The first model focused on migrating fan-shaped cells, which exhibit a typical pattern of actin stress fibre subtypes [30], whereas the second model mainly addressed the recovery of the cells following an acute depletion of myosin II ATPase activity by blebbistatin [49]. Considering other potential assembly mechanisms of dorsal stress fibres and transverse arcs, it is also important to mention the recent study by Nemethova *et al*. [50], which demonstrated filopodia-driven actin stress fibre assembly mechanisms using fish fibroblasts and B16 melanoma cells. Filopodia are rod-like extensions at the leading edge that are composed of a bundle of actin filaments cross-linked by fascin, whereas actin stress fibres are mainly cross-linked by α-actinin. Interestingly, the authors of this study showed that actin filaments generated in filopodia can be ‘re-used’ to assemble lamellar actin stress fibres, which are orientated either perpendicularly or in parallel with the leading edge. In the future, it will be of great interest to investigate these findings in the context of actin stress fibre subtypes. For example, it will be interesting to assess subcellular localization of fascin along actin stress fibre subtypes and possible stress fibre subtype phenotypes in cells deficient in fascin. However, it is of note that in contrast to fibroblasts, migrating U2OS cells do not exhibit evident filopodia structures [30,31].

Ventral stress fibres, which terminate with focal adhesions at both ends, reside on the ventral surface, underneath the nucleus and in the trailing area [29–31,46]. Live cell imaging has revealed that at least a portion of the ventral stress fibres form via the fusion of pre-existing dorsal stress fibres and transverse arcs or in the absence of transverse arcs via the fusion of two dorsal stress fibres [30]. However, it is likely that this fibre subtype could also be assembled via other mechanisms, because mesenchymal-migrating cells lacking dorsal stress fibres still have evident ventral stress fibres [31]. Considering alternative ventral stress fibre assembly mechanisms, the ectopic expression of a formin family member DAAM1 (Disheveled-associated activator of morphogenesis 1) has been shown to enhance ventral myosin IIB-containing actin stress fibres [29]. It is reasonable to assume that ventral myosin IIB-containing actin stress fibres and ventral stress fibres represent the same structures on the basis of their subcellular localization and focal adhesion attachments in U2OS cells [29–31].

## 5. Molecular signature of actin stress fibre subtypes

Distinct actin assembly mechanisms of stress fibre subtypes are intriguing because they suggest molecular and functional differences between the fibre subtypes. Hotulainen & Lappalainen [30] observed that the assembly of dorsal stress fibres occurred via a formin family member mDia1-driven actin nucleation at leading edge adhesions, whereas the Arp2/3 complex was required for the assembly of transverse arcs. Recently, this same research group further showed that the Arp2/3 complex cooperates with mDia2 to assemble transverse arcs [33]. Motivated by these findings, we investigated whether either of the two major non-muscle stress fibre cross-linkers, α-actinin-1 or α-actinin-4, could show any specificity in cross-linking actin stress fibre subtypes [31]. Interestingly, the downregulation of α-actinin-1 resulted in a specific loss of dorsal stress fibres, supporting the existence of distinct molecular signatures in different fibre subtypes [31,47]. Furthermore, the noted dorsal stress fibre phenotype showed a correlation with α-actinin-1 and α-actinin-4 localization along dorsal stress fibres. In dorsal stress fibres, α-actinin-1 is abundant throughout the fibre length, whereas α-actinin-4 accumulates at the base of dorsal stress fibres, elongating from the leading edge focal adhesions [31].

However, how α-actinin-1 and α-actinin-4, which are very similar in their primary structure (87% identity), generate different cross-linking of stress fibre subtypes remains unknown. One possibility involves their less related regions at the N-terminus or in the third spectrin-like repeat, which have been reported to interact with a variety of different molecules, such as CRP1, LPP, Zyxin, CLP-36, RIL or PKN [51–55]. Another possibility involves the regulation of α-actinin-1 and α-actinin-4 binding with filamentous actin. In this respect, the N-terminus of α-actinin-1, which associates with filamentous actin, contains a focal adhesion kinase phosphorylation site (Tyr12) [56,57]. This does not appear to be a major phosphorylation site in α-actinin-4 [58]. Moreover, the phosphorylation of Tyr12 on α-actinin-1 was previously reported to promote tumour cell adhesion in a model in which extracellular pressure was increased in a controlled manner using a gas-based apparatus [56], suggesting a potential dorsal stress fibre-associated function. However, another *in vitro* study demonstrated that Tyr12 phosphorylation in α-actinin-1 decreased α-actinin-1 binding with filamentous actin [57]. Thus, future studies are needed to determine whether Tyr12 phosphorylation of α-actinin-1 is required for dorsal stress fibre assembly.

**Figure 2.**
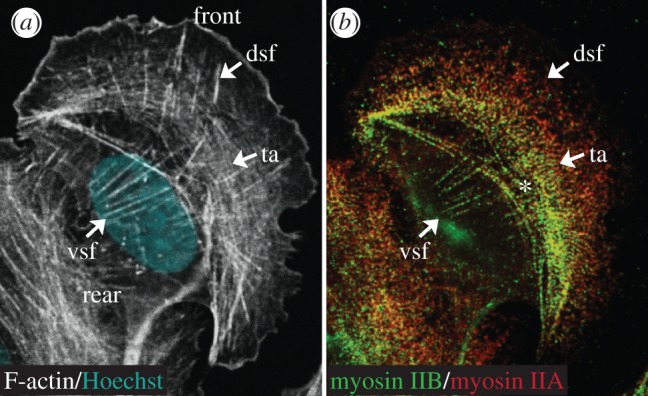
Immunofluorescence images of human osteosarcoma (U2OS) cells reveal a distinct distribution of myosin IIA and myosin IIB on actin stress fibre subtypes. (*a*) A merged image of F-actin (white) and Hoechst (blue) staining; (*b*) a merged image of myosin IIB (green) and myosin IIA (red) staining. Dsf, dorsal stress fibres; ta, transverse arcs; vsf, ventral stress fibres. Note that dorsal stress fibres lack myosin II staining. The asterisk indicates myosin IIB localization on a subset of transverse arcs.

In addition to nucleators and cross-linkers, myosin II motors show interesting stress fibre subtype-specific differences. The most interesting is the lack of myosin II on dorsal stress fibre trunks [31], because it challenges the widely accepted view that all actin stress fibres resemble sarcomere-like contractile structures [28,30,33,59,60]. The non-contractile nature of the dorsal stress fibres has also raised the question of how these cells convey the tension required for the maturation of leading edge adhesions. Although it remains to be elucidated, a likely solution is that this tension is derived from transverse arcs, which directly interact with the elongating dorsal stress fibres [30,31]. Another important myosin II-related observation is that the majority of migrating cells express two out of three non-muscle myosin II isoforms, which exhibit partially overlapping subcellular localization. Previous studies using cell types such as fibroblasts, endothelial cells and melanoma cells have demonstrated that myosin IIA (MHC-IIA, MYH9) localizes predominantly in the anterior portion of migrating cells, whereas myosin IIB (MHC-IIB, MYH10) colocalizes with myosin IIA in the portion of the lamella, and is rich in trailing parts of the cell [61–66]. Thus, these results together with the data obtained on the actin stress fibre subtypes (figure 2) clearly show that myosin IIA decorates both transverse arcs and ventral stress fibres, whereas the ventral stress fibres are rich in myosin IIB. In addition, myosin IIB is detectable on a subset of transverse arcs that are localized closer to the nucleus (figure 2*b*, asterisk). Evidently, myosin II isoforms have a significant contribution to cell migration, and therefore it is tempting to suggest that the myosin II isoform-specific localization reflects stress fibre subtype-specific functions (see §6).

**Figure 3.**
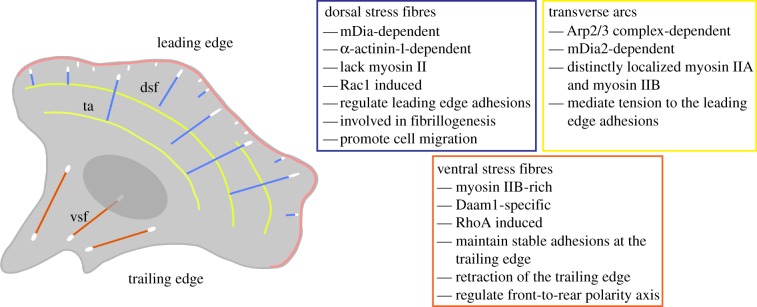
Summary of the molecular differences between actin stress fibre subtypes and subtype-specific cellular activities.

It is also reasonable to assume that the actin filament nucleators and the α-actinin and myosin II isoforms represent only the first molecular signatures between actin stress fibre subtypes. Indeed, some recent studies suggest that the diversity may be greater. For example, different tropomyosin isoforms (Tm1, Tm2/3 and Tm5NM1/2) display stress fibre subtype-specific distributions, particularly along dorsal stress fibres [33]. However, the significance of these tropomyosin isoforms in controlling the function of actin stress fibre subtypes is challenging because the downregulation of a single tropomyosin isoform resulted in a dramatic loss of all actin stress fibres, which suggests that the examined tropomyosin isoforms stabilize actin stress fibres in a non-redundant manner. An exception was tropomyosin 4, which was shown to be required for the recruitment of myosin II to transverse arcs [33]. Other interesting candidate proteins for potential molecular signatures between actin stress fibre subtypes include actin isoforms. We have observed a correlative increase in β-actin at the leading edge of migrating cells in the absence of dorsal stress fibres [31]. Other studies have shown that β-actin-deficient fibroblasts display a severe migration defect, which seems to correlate with an increase in ventral stress fibres [67,68]. In addition, β-actin mRNA levels have been shown to be enriched at the leading edge of migrating cells, and this localization is important for cell migration [69,70]. Furthermore, a recent study demonstrated that β-actin mRNA spent extra time at focal adhesions, which promoted the formation of a stable linkage between adhesions and newly polymerized actin filaments [69]. In the future, it would be of great interest to further study β-actin in the context of actin stress fibre subtypes. The current understanding of molecules that signify actin stress fibre subtypes is summarized in figure 3.

## 6. Functional significance of actin stress fibre subtypes in migrating cells

Mesenchymal cell migration is driven by the assembly of both protrusive and contractile actin filaments [41,42,59]. The major contractile actin assemblies are actin stress fibres and contractile lamellar networks (or contractile-network arrays) [27,49]. Actin stress fibres function in close cooperation with integrin-based adhesions and the ECM, while regulating several cellular functions in migrating cells, such as the maturation of integrin-based adhesions, generation of traction forces [71,72], establishment of the front-to-back polarity axis [29,62,65,73], retraction of the trailing edge [74], mechanotransduction [32,75,76] and ECM remodelling [47,71]. To accomplish directional cell migration, several of these functions need to be regulated in a spatial and temporal manner. An increasing number of studies have proposed that actin stress fibre subtypes could provide this spatial and temporal regulation [28,29,31,32,47] (figure 3). In §6.1, I will discuss in greater detail the current view of stress fibre subtype-specific functions in the context of a migration cycle, which is often used to simplify the complex processes that occur in migrating cells [41,42,59]. However, it is of note that there is a tight interconnectivity between stress fibre subtypes, which is likely to ensure spatio-temporal myosin II-based contractility to achieve directional migration. As an example of this interconnectivity, the formation of ventral stress fibres suppresses protrusion formation at the trailing edge [65,73].

### 6.1. Role of dorsal stress fibres and transverse arcs at the leading edge

The mesenchymal migration cycle can be initiated by various stimuli, such as growth factors, chemoattractants or ECM changes [41,42,59]. Upon exposure to migratory stimuli, cells form a polarized morphology, which is defined as the leading edge extending towards the stimuli (can be either broad lamellipodia or a spike-like filopodia) and a trailing part, which points in the opposite direction (figure 1). Characteristic of the leading edge are actively forming protrusions, which result from actin polymerization and are mainly driven by the activated Arp 2/3 complex, which nucleates the branched actin network at the lamellipodium [42,59]. Subsequently, the formed protrusions are stabilized by maturing adhesion complexes, of which the most well characterized are integrin-based adhesions. This stabilization involves the engagement of the extracellular portion of integrins with ECM proteins, such as fibronectin and collagen, and the interaction of the intracellular portion with actin stress fibres via linker proteins [71,76,77]. Importantly, to fully mature, the integrin-based adhesions require actin stress fibre-mediated tension, [41,42,59,71,76,77], which at the leading edge derives from dorsal stress fibres and at the trailing edge from ventral stress fibres [30,31,46,47]. The maturation of integrin-based adhesions is often described as a continuum of their growth in size. Briefly, short-lived, myosin II-independent nascent adhesions [78] constantly form in the lamellipodia, and a subset of these adhesions grow towards spot-like structures referred to as focal complexes. Upon increased tension, a portion of these focal complexes mature to larger, more elongated focal adhesions [71,76,77]. The necessity of dorsal stress fibres in regulating the maturation of leading edge adhesions is demonstrated in cells that are deficient in either α-actinin-1 expression or mDia activity [31,47]. In these cells, the assembly of dorsal stress fibres is lost, and adhesion maturation becomes impaired, i.e. only nascent adhesions and small focal complexes are detectable. Importantly, in α-actinin-1-deficient cells, trailing edge adhesions mature normally, which strongly suggests that the smaller adhesions sites at the leading edge are due to a lack of dorsal stress fibres and not the lack of α-actinin-1 at focal adhesions [31]. Although the overall functional significance of dorsal stress fibres remains unclear, these fibres have been shown to promote the wound healing rate and cell migration in transwells [31].

From a mechanistic point of view, an interesting open question is how the non-contractile dorsal stress fibres are capable of mediating tension, which is required for adhesion maturation. A likely explanation is that transverse arcs function as a source of myosin II-mediated tension via a direct connection with dorsal stress fibres, which in this model act as straight mediators between the maturating adhesions and transverse arcs. Consistent with this idea, an acute inhibition of myosin II ATPase activity by blebbistatin or an inhibition of ROCK kinase activity by Y-27632 results in the rapid disassembly of transverse arcs (and ventral stress fibres) accompanied by a loss of mature focal adhesions [31,79–81]. Importantly, this occurs over a time-frame when dorsal stress fibres are still present (longer treatment with these inhibitors disassembles all actin stress fibres) [31]. Similarly, the specific loss of transverse arcs via the downregulation of the Arp2/3 complex subunit, p34, appears to induce smaller leading edge adhesions in the presence of dorsal stress fibres [30]. It is tempting to speculate about the potential benefits in regulating leading edge adhesions via the cooperation of dorsal stress fibres and transverse arcs. One notable characteristic of these adhesions is their dynamic nature, in terms of both their rapid turnover rates and their selective maturation, i.e. only a subset of adhesions grow in size [60,71]. Thus, one advantage of this cooperation may be a highly regulated system between the leading lamellipodium and lamella. In this system, adhesions that connect to transverse arcs via dorsal stress fibres can be selected for the maturation process. Conversely, an abrupt disassembly of a dorsal stress fibre dissolves only the attached adhesions, which can induce the rapid and selective turnover of adhesions. Alternatively, additional stimuli (e.g. increased myosin IIB activation along transverse arcs) may result in the formation of ventral stress fibres as a consequence of dorsal stress fibres and transverse fusion, as has been previously observed [30].

### 6.2. Role of ventral stress fibres at the trailing edge

At the same time that dynamic protrusions extend towards the migratory stimuli, the other end of the cell establishes a trailing edge [41,42,59]; this process is referred to as front-to-rear polarity axis formation and is required for directional movement. For example, cells forming randomly located, unstable protrusions lack directionality while migrating [62]. Vicente-Manzanares *et al*. [65] proposed that indeed the important aspect in establishing the trailing edge involves the suppression of protrusions. This suppression was shown to be associated with the formation of stable focal adhesions at the trailing edge, which interact with myosin IIB-containing actomyosin filaments. These filaments are likely to represent ventral stress fibres on the basis of their localization and termination sites. This observation is very interesting because it suggests that the change in molecular composition of actin stress fibres, in this case the recruitment of myosin IIB, plays an important role in regulating the function of the attached adhesions and thereby directing cell migration. Indeed, the well-accepted view is that the adhesion maturation reflects the quantitative differences in protein levels and their phosphorylation status rather than molecularly distinct structures [59]; thus, regulation via attached actin stress fibres could be a likely alternative. Considering the potential mechanisms regarding how myosin IIB stabilizes the trailing edge adhesions, Vicente-Manzanares *et al*. [65] observed that the diphosphorylated mutant of MLC, only in the presence of myosin IIB, strengthened myosin IIB binding to the trailing edge stress fibres and induced more stable adhesions. Furthermore, cells lacking myosin IIB resulted in random protrusions and a loss of directional migration [62,65]. Similarly, this directionality was lost following the depletion of the formin family member DAAM1, which normally induces myosin IIB-enriched ventral stress fibres [29]. However, the remaining unresolved issue is how does myosin IIB stabilize adhesions? One possibility is that myosin IIB-generated tension on ventral stress fibres could be sensed by adhesion proteins, resulting in their activation. Although a direct proof-of-concept remains to be addressed, recent investigations have demonstrated that physical stimuli (such as tension) can activate focal adhesion proteins. For example, the applied mechanical force to talin opens its vinculin-binding site [82] or p130Cas stretching causes its tyrosine phosphorylation [83].

In addition to potential actin stress fibre-mediated suppression of protrusion activities, it is important to mention Rho GTPase Rac. Recently, Rac, which is a widely accepted inducer of protrusions at the leading edge, was shown to be present, but inactive at trailing edge stable adhesions [65]. This was due to a lack of its activation via GEFs (guanine nucleotide exchange factors), such as βPix and DOCK180, which thereby were suggested to be important regulators of adhesion maturation [73]. Taken together, it appears that myosin IIB has a significant contribution in regulating the directional cell migration via ventral stress fibres, whereas myosin IIA is more involved at the leading edge. However, it is of note that whereas myosin IIA is abundantly localized on transverse arcs, myosin IIB is also detectable on a portion of transverse arcs (figure 2). Moreover, myosin IIB downregulation appears to also impair leading edge adhesions [65]. Interestingly, Vicente-Manzanares *et al*. proposed that myosin IIB regulated leading edge adhesions ‘at a distance’ in an indirect manner. In this respect, it is tempting to speculate that the myosin IIB could control the maturation of focal adhesions at the leading edge via attached dorsal stress fibres (figure 2, asterisk).

### 6.3. Other roles of actin stress fibres in mesenchymal-migrating cells

In contrast to amoeboid migrating cells, a characteristic ability of mesenchymal-migrating cells is that they can remodel and degrade the surrounding ECM [34,35]. ECM remodelling, which is best characterized on cells adhering to the fibronectin-rich ECM, is dependent on integrin-dependent adhesions and myosin II-mediated tension [71,84]. On a fibronectin matrix, a subset of focal adhesions undergoes an additional maturation process, which results in fibrillar adhesions. These elongated, α5β1 integrin and tensin-rich stable adhesions are located more centrally when compared with other focal adhesions. A key role of fibrillar adhesions is to assemble fibronectin into fibrils (fibrillogenesis) and thereby remodel the ECM [71,84]. Interestingly, Oakes *et al*. [47] recently showed that in α-actinin-1-downregulated or mDia-inhibited cells, the assembly of dorsal stress fibres was required to form the tensin-rich fibrillar adhesions. This observation suggested a dorsal stress fibre-specific function, which deserves further investigation.

Finally, actin stress fibres are firmly linked to mechanotransduction [32,77,82,83,85]. There is clear correlative evidence between the assembly of actin stress fibres and matrix stiffness, suggesting that the cells can sense the physical properties that surround them. Considering matrix stiffness-sensing and cell migration, a striking observation is the tendency of cells to migrate from a soft matrix to a stiff matrix in the absence of any other migratory stimuli, a phenomenon that has been dubbed ‘durotaxis’ [86]. Until recently, the underlying molecular mechanisms of durotaxis have been uncharacterized. However, Raab *et al*. [87] revealed that the stiff matrix-sensing results in myosin II polarization, i.e. on stiff matrix, diffuse myosin IIA assembles in orientated actin stress fibres, which are then polarized by myosin IIB. This observation is of great interest because durotaxis has been proposed to contribute to wound healing, scarring and the spread of cancer cells, and appears to require actin stress fibres.

## 7. Future perspective

Almost 100 years have passed since the identification of stress fibres. In the past few years, significant progress has occurred in understanding the molecular composition, assembly mechanisms and function of actin stress fibre subtypes in migrating cells. This progress forms a good basis for obtaining a more comprehensive view of these actin-based structures in cell migration. However, major unresolved issues remain, such as the precise function of actin stress fibre subtypes and how the activities of fibre subtypes are interconnected with the achievement of directional cell migration. Interesting interconnectivity candidate regulators include the Rho GTPases Rac1 and RhoA. Rac1 represents a critical regulator of actin polymerization at the leading edge [42,88,89], where it also induces dorsal stress fibres [31]. In contrast, active RhoA promotes contractility and ventral stress fibre formation at the trailing edge [31,59]. In the future, it would be of great interest to study whether reciprocal activation of Rac and RhoA could coordinate the migration cycle at the leading and trailing edges as has been shown in other processes such as a cyclic protrusion activity at the leading edge [90] and an extension and collapse of neurite growth cones [91]. In parallel with Rac and RhoA, key regulators of actin stress fibres include numerous kinases that phosphorylate and activate the myosin light chain of myosin IIA and IIB, such as ROCK-kinases, MLCK, MRCK or NUAK kinases [92–95]. However, the functions of these or other kinases in the context of actin stress fibre subtypes have not been determined yet.

In the future, it is also important to compare actin-based structures and their function in cells migrating on two-dimensional surfaces (e.g. cells grown on plastic or glass) to cells migrating on a three-dimensional environment (e.g. cells embedded in matrigel). It appears that actin-based structures differ substantially depending on the dimensions [37,96–98]. This variation is likely to reflect differences in the physical parameters, such as matrix topography or rigidity. Future comparative two- versus three-dimensional studies could provide hints on how actin-based structures function in different tissue conditions, such as during normal tissue renewal and regeneration or in pathological conditions such as in cancer cell metastasis. Considering these future perspectives, we will need tools to analyse actin stress fibre dynamics on a three-dimensional environment and in tissues. In this respect, interesting tools have been recently generated, including transgenic GFP- and RFP-LifeAct mouse models [99] in combination with advanced intravital imaging methods [100].

Finally, from a technical standpoint, the naming of actin stress fibres and their subtypes requires a consensus within the actin cytoskeleton field. The current variability is highly challenging, e.g. actin stress fibres can be referred to as actin bundles, actomyosin bundles and microfilament bundles, and dorsal stress fibres can be referred to as radial fibres, etc. This creates remarkable challenges in the interpretation of existing data and the discussion of new findings. Hopefully, the progress in understanding the molecular composition and functions of different actin-based structures will help to establish a consensus in actin nomenclature.
